# Comparison of Short-Term Outcomes of DSA and ALMIS Approach for Bipolar Cemented Hemiarthroplasty in Patients with Neck of Femur Fracture

**DOI:** 10.3390/jcm13216465

**Published:** 2024-10-28

**Authors:** Juliane Moussaoui, Jakob Hallbauer, Arne Wilharm, Ivan Marintschev, Gunther Olaf Hofmann, Wolfram Weschenfelder

**Affiliations:** 1 Department of Trauma, Hand and Reconstructive Surgery, University Hospital Jena, 07747 Jena, Germany; juliane.moussaoui@med.uni-jena.de (J.M.); jakob.hallbauer@med.uni-jena.de (J.H.); arne.wilharm@med.uni-jena.de (A.W.); gunther.hofmann@med.uni-jena.de (G.O.H.); 2 Department of Trauma Surgery, Orthopaedics and Spinal Therapy, Catholic Hospital Erfurt, 99097 Erfurt, Germany; ivan.marintschev@med.uni-jena.de

**Keywords:** neck of femur fracture, endoprosthesis, hemiarthroplasty, anterolateral approach, direct superior approach, complication

## Abstract

**Background/Objectives**: A neck of femur (NOF) fracture is one of the most common fractures, and its treatment in the geriatric population using cemented bipolar hemiarthroplasty (HA) is a standard procedure worldwide. Various surgical approaches have been described for this operation, aiming to reduce complications and improve early mobilization. The present study compares two minimally invasive approaches, the anterolateral minimally invasive approach (ALMIS) and the direct superior approach (DSA), with respect to their intraoperative and early postoperative complications in cemented bipolar HA. **Methods**: The medical records of all patients undergoing cemented bipolar HA for a NOF fracture between January 2017 and December 2023 were analyzed. The aim of the study was to compare the two surgical approaches. The evaluation focused on intraoperative parameters and early complications. **Results**: A total of 226 patients were included in the analysis, with 62 undergoing DSA and 164 ALMIS, with an average age of 83.5 years. The two approaches did not differ significantly in terms of stem implantation quality, length of hospital or intensive care unit stay, postoperative mobilization, or the need for transfusions. However, the ALMIS was associated with a significantly shorter operative time (DSA: 89.7 min vs. ALMIS: 77.2 min; *p* < 0.01). On the other hand, the DSA had a significantly lower complication rate (DSA: 0/61 vs. ALMIS: 11/163; *p* = 0.04). **Conclusions**: In a comparison of the two minimally invasive approaches, DSA and ALMIS, for treating a NOF fracture with cemented bipolar HA, the DSA demonstrated a lower complication rate, though it had a slightly longer operating time.

## 1. Introduction

Neck of femur (NOF) fractures are a significant health problem in the elderly population, associated with high morbidity and mortality rates, and they significantly impact the quality of life of affected individuals [1]. Hemiarthroplasty (HA) is the standard treatment for these fractures in the geriatric population. The primary aim of this treatment is postoperative remobilization with full weight bearing to avoid complications associated with immobilization [2]. Additionally, the chosen procedure should have a low risk of complications and the need for revisions.

Various surgical approaches to the hip have been described in the literature for performing total hip arthroplasty (THA) or hemiarthroplasty (HA) effectively. These include conventional approaches and their modifications, as well as soft-tissue-sparing, minimally invasive approaches that promote early mobilization [3,4].

A modification of the anterolateral approach is the anterolateral minimally invasive approach (ALMIS), which is performed through the muscle interval between the tensor fasciae latae anteriorly and the gluteus medius posteriorly, with an anterior capsulotomy. The capsule is usually resected and not reconstructed at the end of the operation. Shigemura et al. demonstrated in a meta-analysis that the functional outcomes of ALMIS are superior to those of the classic lateral approach, though it is associated with higher intraoperative blood loss [5]. In a retrospective analysis by de Jong et al., ALMIS showed a shorter operative time compared to the classic anterolateral approach, with similar complication risks [6]. Tsailas et al. compared ALMIS to the conventional posterior approach and found fewer postoperative leg length discrepancies, although it resulted in a longer operative time [7].

The direct superior approach (DSA) is a minimally invasive variant of the posterolateral approach (Kocher-Langenbeck) that enters the joint posterior to the gluteus medius. The capsulotomy is, therefore, performed dorsally and is typically reconstructed at the end of the procedure. Ulivi et al. demonstrated better postoperative function with the DSA compared to the classic posterolateral approach, though it was associated with an increased operation time in total hip arthroplasty (THA) [8]. This increase in operative time was also reported by Duijnisveld et al. [9]. Leonard et al. found a shorter hospital stay in patients undergoing a THA via the DSA compared to the standard posterior approach [10], while Nam et al. did not find a difference in pain levels between patients who had a THA through either approach [11]

In summary, there appears to be no definitive benefit of any conventional approach for hemiarthroplasty (HA) [12,13], although some advantages have been observed with minimally invasive approaches [14,15,16,17]. While studies have compared conventional approaches to minimally invasive ones, none have directly compared two minimally invasive techniques. Therefore, the aim of this study is to compare the early complications in patients undergoing HA through either the direct superior approach (DSA) or the anterolateral minimally invasive approach (ALMIS). The study considers the occurrence of relevant operation-associated complications as a composite outcome.

## 2. Materials and Methods

### 2.1. Study Population

**Figure 1:**
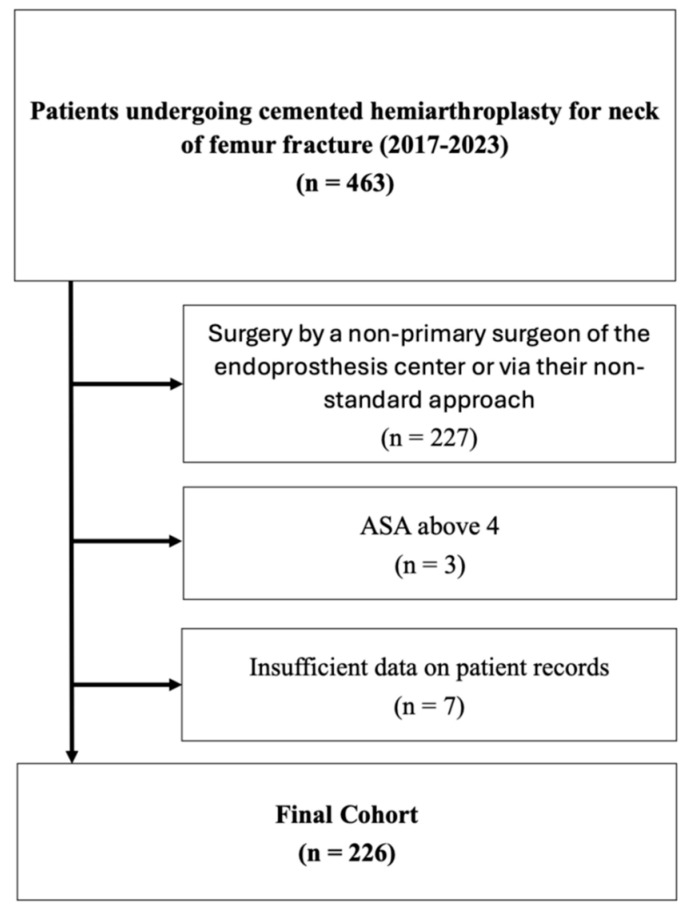
Flowchart of study population.

We retrospectively reviewed the records of patients who underwent surgical treatment with bipolar hemiarthroplasty for a neck of femur fracture between 2017 and 2023 at our institution. During this period, 463 patients received a cemented hemiarthroplasty for a neck of femur fracture at our institution. Of these, 236 patients met our inclusion criteria, while 10 had to be excluded (see Figure 1). Ethical approval for the study was obtained from the local ethics committee.

### 2.2. Inclusion Criteria

- Neck of femur (NOF) fracture treated with cemented hemiarthroplasty (HA) between 2017 and 2023;
- Surgery performed by a Consultant Orthopaedic Surgeon certified as the main surgeon of the certified endoprosthesis center, using the surgeon’s standard approach (either DSA or ALMIS).

### 2.3. Exclusion Criteria

- American Society of Anesthesiologists (ASA) classification score greater than 4;
- Insufficient data in patient records.

### 2.4. Data Collection and Statistical Analysis

Patient characteristics (age, gender, and BMI), medical history (ASA, anticoagulation, musculoskeletal disorders, diabetes mellitus II, or osteoporosis), laboratory results (pre-/postoperative hemoglobin), and imaging findings were collected from patient records. Postoperative imaging assessments, including stem position, quality of cementation, avulsion of the greater trochanter, and other periprosthetic fractures, were independently performed by two experienced orthopedic surgeons. Any disagreements were resolved through discussion and consensus with a third reviewer. Cementation quality was graded using the classification proposed by Barrack et al. [18,19]. The actual intraoperative blood loss was estimated using the formulas of Nadler et al. and Good et al. [20,21]. Hemiarthroplasty was performed by an experienced arthroplasty surgeon (certified as the main surgeon of the endoprosthesis center) via either the direct superior approach (DSA) or the anterolateral minimally invasive approach (ALMIS). Postoperative management was standardized for all patients. The implants used were either the cemented Twinsys© stem (Mathys, Bettlach, Switzerland) or the cemented MV40© stem (Stryker, Kalamazoo, MI, USA) with a bipolar head. The wound was closed following our hospital’s internal protocol, using staples without the routine use of drains. Provided there were no other indications for anticoagulation, patients received low molecular weight heparin prophylactically during the postoperative period. Six weeks postoperatively, patients were reviewed either in the clinic or via phone contact with their respective general practitioners.

Statistical analysis was performed using SPSS version 27.0 (IBM©, New York, NY, USA). All patients that met the inclusion criteria and none of the exclusion criteria were included in the analysis. Categorical data were compared using the Chi^2^ test. Since the continuous data did not follow a normal distribution, non-parametric tests were used to identify differences; the Mann–Whitney U test was applied for comparisons between two groups, and the Kruskal–Wallis test for comparisons among more than two groups. The mean and standard deviation are reported in the text. For any significant differences, an effect size (Cohen’s d) was calculated for metric variables. A *p*-value of less than 0.05 was considered statistically significant.

## 3. Results

### 3.1. Study Population

The study population comprised 138 females and 88 males, with ages ranging from 57 to 100 years and a mean age of 83.5 years (standard deviation SD 7.4 years). There was a significant age difference between sexes (*p* = 0.03), with men averaging 81.8 years and women averaging 84.6 years. Of the patients, 62 were operated on using the direct superior approach (DSA) and 164 using the anterolateral minimally invasive approach (ALMIS). The mean BMI was 24.9 kg/m^2^ (SD 4.5 kg/m^2^). No significant differences were found between the approaches regarding age, gender, BMI, ASA classification, side, implant type, preoperative anticoagulation, musculoskeletal diseases, osteoporosis, or preoperative type II diabetes mellitus (see Table 1).

### 3.2. Results

**Figure 2:**
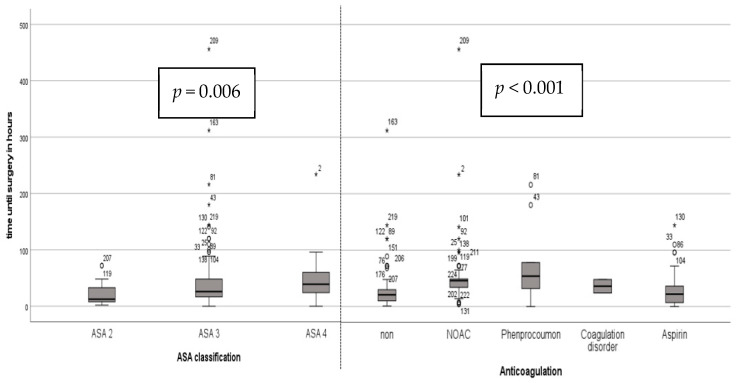
Time until surgery depending on ASA classification and preoperative anticoagulation.

The average time from presentation to surgery was 40 h (SD 49 h), with 50.8 h (SD 75 h) in the DSA group and 36.3 h (SD 36 h) in the ALMIS group. This difference was not statistically significant (*p* = 0.45). A clear correlation was observed between the time delay to surgery and ASA classification (*p* = 0.01) as well as preoperative anticoagulation (*p* < 0.01; see Figure 2). For patients with ASA 2, the average time until surgery was 21.5 h (SD 20 h), compared to 41.6 h for ASA 3 (SD 51 h) and 53.4 h for ASA 4 (SD 58 h). Patients not on anticoagulation were operated on average 29 h after presentation (SD 39 h), those on new oral anticoagulants (NOACs) after 55 h (SD 61 h), patients on phenprocoumon after 76 h (SD 69 h), those with coagulation disorders after 36 h (SD 20 h), and patients on aspirin after 31.5 h (SD 36 h).

**Figure 3:**
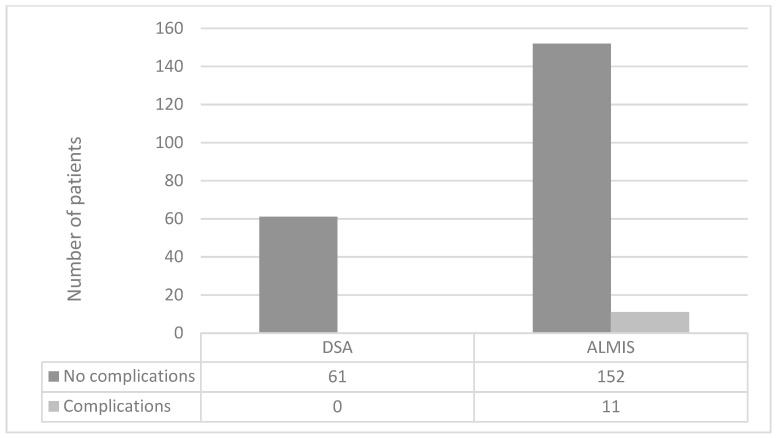
Patients with complications excluding in-house death.

The analysis of individual complications and postoperative outcomes revealed no significant differences between the two approaches concerning avulsion of the greater trochanter (GT), other periprosthetic fractures, shaft alignment, quality of cementation, postoperative wound healing problems/infections, dislocation of the hemiarthroplasty (HA), degree of mobilization, or in-house death (see Table 2). However, when combining cases with relevant operation-associated complications (including GT avulsion, other periprosthetic fractures, wound healing problems/infections requiring revision, and HA dislocation), a significant difference was found in favor of the DSA approach (*p* = 0.04; see Figure 3).

**Figure 4:**
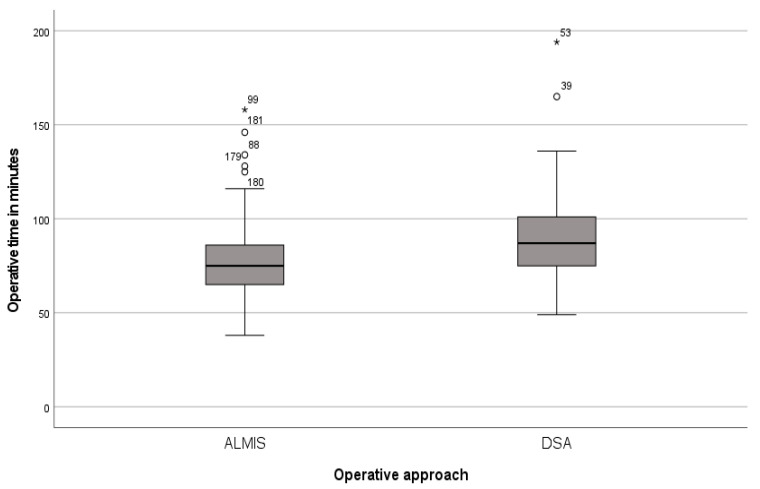
Operative time for different approaches.

The results for the metric variables assessed in the study are presented in Table 3. There was a significant difference between the two approaches in terms of operative time, defined as the time from incision to closure (*p* < 0.01; Cohens’ d = 0.59; see Figure 4), and the pre- to postoperative change in hemoglobin levels (*p* = 0.03; Cohens’ d = 0.23). However, the estimated intraoperative blood loss, calculated using the methods of Nadler et al. and Good et al., showed no significant difference between the approaches (*p* = 0.72 and *p* = 0.31, respectively). Additionally, the study examined potential correlations between preoperative hemoglobin levels and the pre- to postoperative change in hemoglobin with pre-existing use of anticoagulants. No significant correlations were found for preoperative hemoglobin (*p* = 0.31), the pre- to postoperative hemoglobin difference (*p* = 0.22), or the estimated intraoperative blood loss according to Nadler et al. and Good et al. (*p* = 0.50 and *p* = 0.55, respectively).

## 4. Discussion

Our study is the first to compare the two minimally invasive approaches, DSA and ALMIS, for the implantation of a cemented bipolar hemiarthroplasty in the treatment of neck of femur fractures.

The results indicate a significant delay in time to surgery for patients with higher ASA scores and those on pre-existing anticoagulant therapy. This delay is associated with increased mortality in these geriatric patients [1]. Currently, the American Heart Association recommends deferring non-urgent surgery for patients on NOACs for at least 48 h after the last dose, particularly for operations with a high risk of bleeding [22]. However, the greatest delays were observed in patients taking phenprocoumon. This delay is primarily due to the fact that many of these patients were anticoagulated for mechanical heart valve replacements, making it challenging to reverse the anticoagulation quickly without the use of prothrombin complex concentrate (PPSB).

The data regarding postoperative outcomes showed no significant differences between the two approaches in terms of stem position, quality of cementation, and early postoperative mobilization. Leonard and Ohly found similar results when comparing the DSA approach to the posterior approach, with no differences in stem alignment or patient satisfaction [10]. The statistically significant lower rate of intra- and postoperative complications (such as wound healing disturbances, GT avulsion, and dislocation) associated with the DSA approach is one of the main findings of this study. Tsailas et al. did not find this difference when comparing the ALMIS with the conventional posterior approach [7]. The higher incidence of GT avulsions with lateral and anterolateral approaches is a well-documented issue, as noted by Khan et al. in their review comparing these approaches to the direct anterior approach (DAA) [16]. The increased rate of GT avulsions in the ALMIS group compared to the DSA group may be related to the stem implant used (a type 3 design with a line-to-line principle), which was the same for both approaches, as well as the specific characteristics of the approach. The stem is relatively bulky and must be inserted straight into the medullary canal, potentially generating higher tensions during implantation via the anterolateral approach compared to the dorsal approach. This issue might be mitigated with a different implant design, such as a banana-shaped or calcar-guided stem, which could allow for around-the-corner implantation. Dislocations might be attributed to the capsular repair in the DSA approach compared to the capsular resection in the ALMIS approach. A clear explanation for the higher revision rate due to wound healing or infection could not be found in the existing literature. In the ALMIS group, more patients with diabetes mellitus were operated on; though this difference was not significant, it could have potentially influenced the infection rate. Additionally, no data were available on pre-existing ulcers on the operated extremities, which might have affected the risk of infection.

In our study, the ALMIS approach demonstrated a significantly shorter operative time compared to the DSA approach. The observed effect size of Cohen’s d = 0.59 indicates a medium effect. Similarly, Duijnisveld et al. reported a longer operating time for the DSA compared to the mini-posterior approach, and Ulivi et al. found a longer time for the DSA compared to the conventional posterior approach [8,9]. However, it is worth noting that posterior approaches are associated with an increased dislocation rate in hemiarthroplasty, a disadvantage that was not observed with the DSA in our study [23,24]. The ALMIS approach also showed a shorter operative time compared to the conventional anterolateral approach [6]. In our study, the longer operative time associated with the DSA did not affect survival rates or the length of ICU or hospital stays.

Our study found a significant difference in intraoperative blood loss when comparing the change in hemoglobin levels from preoperative to postoperative day. However, there was no significant difference between the two approaches when using the formulas by Nadler et al. and Good et al., which account for gender, BMI, and blood transfusion administration. Ulivi’s study on the DSA approach compared blood loss with the conventional posterior approach and demonstrated lower blood loss with DSA [8]. This suggests that minimally invasive approaches may be associated with less overall blood loss.

### Strength and Limitations

One of the strengths of this study is that it is the first to compare the two minimally invasive procedures, ALMIS and DSA, for hemiarthroplasty. All patients were treated in the same hospital, following the same standards, and by the same team, minimizing potential sources of error.

However, the retrospective design of the study is a limitation. Despite this, randomization was somewhat inherent, as acute patients were assigned to the operating list of available surgeons at random. Due to the retrospective nature of the study, patient-reported outcome measures could not be collected, which might have provided additional insights into actual postoperative function.

## 5. Conclusions

Compared to ALMIS, the DSA approach is associated with fewer intraoperative and postoperative complications and a tendency towards reduced blood loss in the treatment of patients with femoral neck fractures using bipolar hemiarthroplasty. However, the average operative time for DSA is approximately 12.5 min longer. In our study, this increased operative time did not affect survival rates or the length of ICU or hospital stays. At this time, the authors cannot make a clear recommendation for one approach over the other and use both in their clinical practice, depending on specific needs.

## Figures and Tables

**Table 1:** Basic preoperative patient data.

		DSA	ALMIS	*p*
Age in Years		84 (SD 8.0)	83.3 (SD 7.2)	0.438
Gender	male	28 (45%)	60 (37%)	0.238
	female	34 (55%)	104 (63%)	
ASA	2	3 (5%)	25 (15%)	0.101
	3	54 (87%)	129 (79%)	
	4	5 (8%)	10 (6%)	
BMI	mean in kg/m^2^	24.2 (SD 3.9)	25.2 (SD 4.6)	0.103
Side	right	25 (40%)	82 (50%)	0.194
	left	37 (60%)	82 (50%)	
Implant	Mathys	46 (74%)	127 (77%)	0.607
	Stryker	16 (26%)	37 (23%)	
Anticoagulation	non	31 (50%)	74 (45%)	0.767
	NOAC	16 (26%)	53 (32%)	
	Phenprocoumon	3 (5%)	8 (5%)	
	Disorder	0 (0%)	2 (1%)	
	Aspirin	12 (19%)	27 (17%)	
Musculoskeletal disorders	No	61 (98%)	153 (93%)	0.128
	Yes	1 (2%)	11 (7%)	
Osteoporosis	No	55 (89%)	128 (78%)	0.068
	Yes	7 (11%)	36 (22%)	
Diabetes mellitus II	No	49 (79%)	114 (70%)	0.325
	Yes	13 (21%)	49 (30%)	

**Table 2:** Postoperative outcome and complications (* indicating *p* < 0.05).

		DSA	ALMIS	*p*
GT avulsion	No	61 (100%)	158 (97%)	0.167
	Yes	0 (0%)	5 (3%)	
Other periprosth. fracture	No	61 (100%)	163 (100%)	n/a
	Yes	0 (0%)	0 (0%)	
Shaft alignment	Varus	9 (15%)	17 (11%)	0.636
	Parallel	33 (54%)	96 (59%)	
	Valgus	19 (31%)	49 (30%)	
Quality of cementation	A	27 (45%)	94 (58%)	0.250
	B	17 (29%)	43 (26%)	
	C	8 (13%)	12 (7%)	
	D	8 (13%)	14 (9%)	
Revision due to wound healing or infection	No	61 (100%)	157 (96%)	0.129
	Yes	0 (0%)	6 (4%)	
Dislocation of bipolar head	No	61 (100%)	161 (99%)	0.385
	Yes	0 (0%)	2 (1%)	
Degree of mobilization	Only bed	6 (11%)	21 (14%)	0.839
	Few steps	23 (40%)	54 (37%)	
	At ward level	28 (49%)	71 (49%)	
Death during hospital stay	No	57 (92%)	154 (94%)	0.596
	Yes	5 (8%)	10 (6%)	
Combined complications excl. death	No	61 (100%)	152 (93%)	* 0.037
	Yes	0 (0%)	11 (7%)	

**Table 3:** Metric parameters including length of stay, operative time, and blood loss (* indicating *p* < 0.05.

	DSA	ALMIS	*p*
Length of hospital stay in days	11.3 (SD 6.3)	11.2 (SD 5.3)	0.411
Length of stay in ICU in days	0.5 (SD 1.3)	0.5 (SD 2)	0.190
Operative time in minutes	89.7 (SD 25.6)	77.2 (SD 19.1)	* <0.001
Setup time in minutes	36.7 (SD 14.1)	32.6 (SD 10)	0.063
Preoperative hemoglobin in mmol/L	7.4 (SD 1.3)	7.6 (SD 1.0)	0.169
Postoperative hemoglobin in mmol/L	6.0 (SD 1.0)	6.0 (SD 0.9)	0.528
Pre- to postoperative difference in hemoglobin in mmol/L	1.4 (SD 1.0)	1.6 (SD 0.8)	* 0.034
Number of transfusions in units	0.3 (SD 0.9)	0.1 (SD 0.4)	0.181
Blood loss according to Nagler in dL	42.67 (SD 8.31)	42.74 (SD 7.21)	0.716
Blood loss according to Good in L	1.04 (SD 0.74)	1.08 (SD 0.58)	0.313

## Data Availability

The data presented in this study are not publicly available but available on request from the corresponding author. The data are not publicly available due to privacy and ethical restrictions.
